# Endemic Cyprus Scops Owl *Otus cyprius* Readily Breeds in Artificial Nest Boxes

**DOI:** 10.3390/ani11061775

**Published:** 2021-06-14

**Authors:** Savvas Iezekiel, Reuven Yosef, Constantinos Themistokleus, Dimitrios E. Bakaloudis, Christos G. Vlachos, Andreas Antoniou, Eandas Iezekiel, Malamati A. Papakosta, Jakub Z. Kosicki

**Affiliations:** 1 Department of Forests, Ministry of Agriculture, Rural Development and Environment, Nicosia 1414, Cyprus; iezekiel@cytanet.com.cy; 2 Ben Gurion University of the Negev—Eilat Campus, P.O. Box 272, Eilat 88000, Israel; 3 Department of Forests, Ministry of Agriculture, Rural Development and Environment, Kedron 1 str., Panagia 86400, Cyprus; cthemistokleous@fd.moa.gov.cy; 4 School of Forestry and Natural Environment, Aristotle University of Thessaloniki, P.O. Box 241, 541 24 Thessaloniki, Greece; debakaloudis@for.auth.gr (D.E.B.); cvlachos@for.auth.gr (C.G.V.); 5 Cyprus Association for the Protection of Avifauna Ioanni Kapodistria 41A, Nicosia 2321, Cyprus; andrantoni@gmail.com (A.A.); Eandas_iezekiel@hotmail.com (E.I.); 6 Department of Forestry and Management of the Environment & Natural Resources, Democritus University of Thrace, 68200 Orestiada, Greece; mpapakos@fmenr.duth.gr; 7 Department of Avian Biology and Ecology, Faculty of Biology, Adam Mickiewicz University, Poznań, ul. Uniwersytetu Poznańskiego 6, 61-614 Poznań, Poland; kubako@amu.edu.pl

**Keywords:** Cyprus, island endemic, Scops Owl, *Otus cyprius*, nest box

## Abstract

**Simple Summary:**

Nest boxes are considered a valid conservation tool in order to sustain wild populations of birds. The Cyprus Scops Owl was recently defined as a new species. However, the extant information on the species is sporadic and the population level is not confirmed. In order to evaluate the ability of the species to use nest boxes, and to understand its habitat preferences, we placed boxes in rural areas, at the forest edge, and in the Paphos Forest. We found that the Cyprus Scops Owl displayed a preference for the forest edge and rural areas; although we also had, several pairs occupy nest boxes in the forest. We show that the nest box strategy can be implemented if in the future the species is threatened.

**Abstract:**

As is well-known, endemic island bird species are especially vulnerable to extinction from anthropogenic environmental change and reduced fitness compared with mainland taxa. The Cyprus Scops Owl, *Otus cyprius,* is a recently recognized island endemic species whose ecology and breeding biology have not been studied. It nests mainly in holes in trees and buildings, so the felling of old trees, modern architectural practices, and the renovation of old houses in villages may reduce nest site availability. Its population trend is also unknown. Therefore, to better determine its ecological requirements and habitat preferences we placed nest boxes in rural areas adjacent to the forest, in the forest, and in the ecotone between them, and used breeding success as our indicator of habitat suitability. We found that breeding parameters like laying date, clutch size, length of the incubation period, hatching day, hatching success, and number of nestlings did not differ between the three habitats. Despite the low level of nest box occupancy rate (5–11%) the endemic Cyprus Scops Owl readily breeds in artificial nests. Therefore, although we are unaware of any current threats to the Cyprus Scops Owl, we recommend that its conservation be prioritized, including studies, monitoring, habitat conservation, and the provision of nest boxes.

## 1. Introduction

Natural cavity-nesting animals that roost or breed use holes in buildings or human-made constructions like nest boxes [1]. Nest boxes can either supplement existing natural cavities or replace them when destroyed and can be a technique in the toolbox of conservation in order to recover declining populations (e.g., Lesser Kestrel, *Falco naumanni*; [2]). Some countries, such as Germany [3] and Poland [4], have legislated rules that require homeowners to install nest boxes as compensation for destroyed nest sites.

The use of nest boxes has allowed the study of life history traits and strategies of many cavity-nesting species by allowing easier access for monitoring and handling of the study organisms [5]. Nest boxes also help control stochastic events, thereby enhancing the chances of survival by artificial, conservation oriented intervention [6]. However, in sensitive species (declining populations, endemics, etc.) it is advisable to test the efficacy of this technique while viable populations still exist in the wild (e.g., [7]). Strigiformes is an avian order that readily occupy nest boxes (e.g., *Tyto alba* [8], *Aegolius funereus* [9,10], *Athene noctua* [6], *Otus scops* [11]).

One of the smallest owls of the Strigidae family is the Scops Owl (genus *Otus*). A recently recognized species is the island-endemic Cyprus Scops Owl (*O. cyprius*; [12,13,14,15]).

The endemic species is presently considered to be of ”Least Concern” by the International Union for Conservation of Nature (IUCN) Red List, with a breeding population of 10,000–24,000 individuals and its current population trend unknown [16]. The local status of this species is considered as common, mainly resident and breeding in villages, lightly wooded areas, and open pine forest up to 1900 m ([17,18,19,20] Cyprus Bird Reports). Hadjisterkotis [21] discovered that they also use nest boxes and recommended their widespread provision because of the frequent felling of the very old forest trees that develop natural cavities. This, combined with the fact that Cyprus has no woodpeckers to create holes in the forest trees, and that the most common forest tree, the Calabrian pine (*Pinus brutia*), rarely develops cavities until it is old, emphasizes the importance of leaving these old trees unfelled.

Owls are affected by edge-effects of transition zones between two habitats [22]. In our case, these are the transitions from rural areas to the Paphos Forest. Therefore, we set up an experimental design and placed nest boxes in rural areas adjacent to the forest, on trees in the ecotone at the forest line, and in the forest to understand the ecological requirements of the endemic Cyprus Scops Owl. We used breeding success and density of occupied nest boxes as our indicators of habitat suitability and hoped to understand which of the three aforementioned habitats best suited the study species. We expected to find that rural areas would have the highest levels of nest loss owing to predation (e.g., domestic cats *Felis catus,* [23]), while the forest edge would have the lowest occupancy and nesting success (cf. [22,24]). We considered the forest to be the optimal habitat, which would allow for the safest, most successful colonization of the nest boxes. We reasoned that if the species can breed in artificial nests independent of habitat type, further anthropogenic-related changes of Cyprus would have less impact on the population of this endemic species.

## 2. Material and Methods

**Figure 1:**
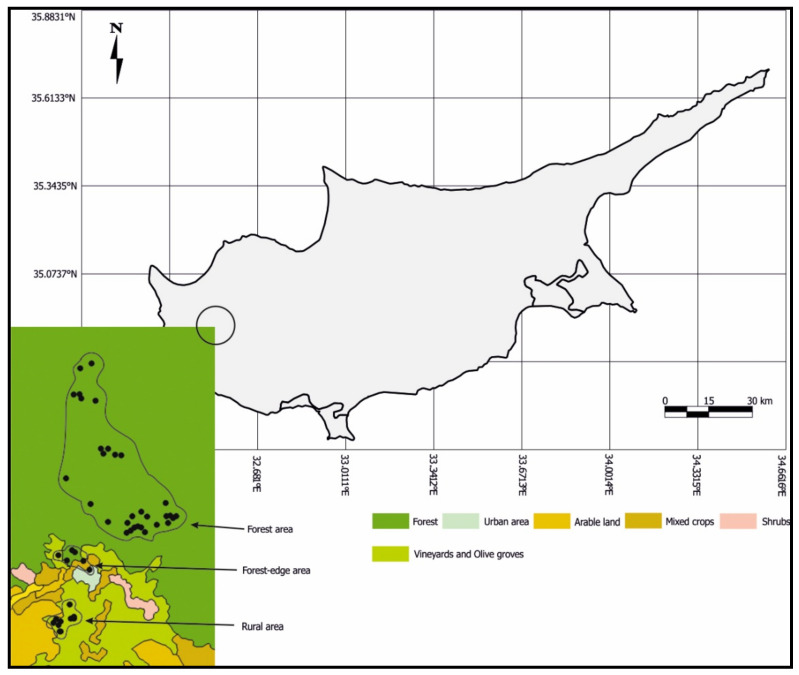
Map showing the location of Paphos Forest (circle) on the Island of Cyprus and the locations of the nest boxes in the rural, forest-edge, and forested areas.

Our study was conducted in the Paphos Forest Reserve, an area of 62,000 ha, which is situated within the Troodos massif in the west of the Republic of Cyprus (Figure 1), and ranges from sea level to >1300 m above sea level. It is a typical Mediterranean forest with coniferous (Calabrian pine, Cyprus cedar *Cedrus brevifolia*) and broadleaf trees (Golden oak *Quercus alnifolia*, Planetree *Platanus orientalis*). The Calabrian pine is the predominant forest tree in our study area; with a closed canopy and an understory with dense low maquis shrubs (e.g., *Cistus creticus*). In this area, between 2015 and 2018 we placed a total of 238 nest boxes (Figure S1). Of these, 91 were placed in rural areas/traditional villages on houses or walls (2.5 km^2^, density 36.4/km^2^), 34 on trees in the transition zone between the villages and the forest (the ecotone, 2 km^2^, density 34.0/km^2^), and 113 in the forest (12.2 km^2^, density 9.26/km^2^). The altitude above sea level ranged from 525 m in the lowest box to 1015 m in the highest. In the rural areas, nest boxes were placed at an average height of 3.5 m (±1.2), in the ecotone and the forest, most nest boxes were placed on *Pinus brutia* (56, 85%) at an average height of 5.8 m (±1.5) and depended on tree availability.

We considered a nest box as occupied if we observed both adults entering it. All nests included in the study were observed periodically with the help of a camera mounted on a telescopic pole. We observed all breeding pairs weekly through the complete breeding cycle from early April to early June.

We analyzed breeding parameters, such as laying date, incubation period (from first day of incubation to hatching date), hatching date, clutch size (number of eggs in nest), number of eggs hatched, and length of breeding attempt (from laying of the first egg to fledging of the last young). Laying and hatching dates were expressed as Julian Days. Owing to the loss of nestlings, we separated the number of hatched eggs from the number of nestlings that subsequently fledged. We considered that Scops Owl females lay one egg every 24 h, usually at dawn. Scops Owl is known to be an ultimate brooder [25,26]. Based on this we established the laying dates and brooding periods for clutches laid in between visits using the Mayfield method [27,28], wherein the median day between visits was considered to be the day of laying, hatching or fledging. The number of nestlings was calculated based on the inspection after hatching. A reproductive attempt was considered successful if at least one young fledged. The breeding success was defined as the proportion of successful nests to all inspected nests and allowed us to compare the breeding performance between the three kinds of artificial nest sites. Because these parameters could be influenced by year, we performed Factorial ANOVA where year (2015–2018) and habitat type for nest boxes (rural, forest, forest edge) were considered as factors [29]. Bonferroni corrections were applied to adjust the alpha values for the increased probability of obtaining statistical significance from multiple testing. Not all information was obtained for all nests studied and resulted in differences in sample sizes between the different analyses. The Kruskall-Wallis test with Dunn’s test [30] as a post-hoc was employed to analyze density differences between years. Mean values are presented with 95% confidence limits (CL) or standard deviation (SD).

## 3. Results

Of 238 nest boxes available during the four breeding seasons of 2015–2018 in three habitats, 91 (38%) in total were occupied, i.e., 9.5% per year, of the 91 nest boxes placed in the rural areas, an average of 6 (6.6%) were occupied per year (in total, 24 nest boxes were occupied i.e., 26.4%), of 34 placed at the forest-edge 5.25 (15.4%) per year were occupied (20, 62%), and of 113 boxes placed in the forest, an average of 11.75 (10.3%) were occupied per year (47, 42%). However, these differences were not significant (chi-square = 5.89, df = 2, *p* = 0.052). The mean (±SD) density of occupied nest boxes per year for rural, forest-edge, and forest were respectively: 2.4 (±0.56), 2.62 (±0.81), 0.96 (±0.33)/km^2^. Although, these differences were significant (Kruskal-Wallis test: H = 7.59, df = 2, *p* = 0.02), according to Dunn’s test, the difference was only between forest and forest-edge (Z = −2.55, *p* = 0.031, in other cases, *p* for Dunn’s test > 0.05).

The occupancy rate of the artificial nest sites was 6.3% (CL: 2.9–9.8) in 2015, 8.4% (CL: 2.9–9.6) in 2016, 11.7% (CL: 2.9–16.1) in 2017, and 10.5% (2.8–16.4) in 2018, and did not differ between the years (Chi-square = 1.53, *p* = 0.64). The laying date, clutch size, incubation period, hatching day, number of hatchlings, and number of fledglings did not differ between the artificial nest sites located in three different places (Factorial ANOVA; laying dates: F_3, 87_ = 1.52, *p* = 0.211; clutch: F_3, 87_ = 1.42, *p* = 0.233; incubation: F_3, 74_ = 0.696, *p* = 0.556; hatching: F_3, 74_ = 1.41, *p* = 0.232; eggs hatched: F_3, 87_ =1.56, *p* = 0.203; fledglings: F_3, 87_ = 1.47, *p* = 0.227; Table 1). Furthermore, overall breeding success was 84.8% (N = 92); with 80.8% in the forest, 95.2% at the forest edge, and 83.3% in rural areas and did not differ between years (chi-square = 0.67, df = 2, *p* = 0.71).

## 4. Discussion

We found that the endemic Cyprus Scops Owl will breed in artificial breeding boxes and that the technique can be an alternative solution for the protection of endemic species in highly exploited habitats. This finding corresponds with other studies, which stress that nest boxes ensure the survival of the bird population where nesting habitat is a limiting factor (cf. [31]). This conservation tool has been successfully used in other species [24,32,33,34,35]. However, no previous studies have evaluated the viability of nest boxes for Cyprus Scops Owl. Hence, the importance of this study is in verifying the hypothesis that this endemic species will breed in nest boxes independently of habitats in a rapidly changing island landscape.

We did not find any differences in the phenology and the basic reproductive parameters between the nest boxes located in three habitats and do not substantiate our hypothesis. We had predicted that we would find increased predation, and hence lower breeding success, in rural areas and at the forest edge, and expected the forest to be the optimal habitat with the highest density and breeding success. Although breeding success was indeed highest in the forest and lowest in the rural area, the differences are not significant. However, one must take the evaluation of breeding success with care because it could be similar in habitats with different “quality” if, for example, a lower owl density balances the lower availability of food in the poorer habitat. However, occupancy rate can also suggest preference by the studied organism. In the Cyprus Scops Owl, occupancy was significantly higher at the forest-edge than in the forest, taking intermediate values at the rural areas. We think that this preference is because of the foraging opportunities offered by the open spaces at the forest-edge and in rural areas, which facilitate prey detection and increased foraging success (cf. [36]).

The reproductive output of the Cyprus Scops Owl (83.3%, N = 91) was similar to other studies, but we were surprised to find that relatively few studies report breeding success in the nominate Eurasian Scops Owl *O. scops* in Europe. Bavoux et al. [37] reported breeding success of 64% (N = 142) on Ile d’Oleron (Charente-Maritime, France) and claimed that the loss of wooded areas and decline in insect abundance because of the reduction in cultivated areas may have affected the species. Blanco et al. [38] found breeding success to be 69% (N = 32) in Spain, but did not study the environmental effects on breeding success. Toyama et al. [39] reported breeding success of 96% (N = 53) in the Japanese Scops Owl (*O. semitorques*) and 77% in the Ryuku Scops Owl (*O. elegans*; N = 150) on the island of Okinawa and considered predation to be the main factor affecting breeding success. Furthermore, on the island of Minami-Daito, in the northwest Pacific Ocean, breeding success of the Daito Scops Owl (*O. e. interpositus*) was 79% (N = 95; [40]), while only 25% (N = 8) in the Seychelles Scops-Owl (*O. insularis*; [41]), where alien predators were considered the main cause. The latter three are of interest because they are all island endemics, like our Cyprus Scops Owl, displaying low breeding success owing to either high predation levels or habitat change. However, the differences in occupancy could also reflect differences in nest-hole availability between different habitats.

We speculate that at present the Cyprus Scops population is stable with high reproductive output when compared to other studies. Other factors influencing the evolution of the population may have been the absence of native mammalian predators [42] or Tawny Owls *Strix aluco* [43], the latter of which are significant predators of *O. s. scops* elsewhere [44], probably resulting in reduced predation and higher breeding densities compared to the mainland. However, one must take into account that all of the above mentioned studies, including ours, did not evaluate the rate of loss of holes due to building restoration, or logging, or hole-availability in their respective study areas. Also, in comparisons between habitats or study areas there is no data on hole availability or density, prey type and density, tree and hole preferences, other avian species densities, and other possible perturbations that could influence nest-site selection, and should be included in future studies.

In Northern Italy, agricultural intensification in the form of vineyards and in the intensive use of pesticides was probably responsible for the decline of the Eurasian Scops Owl. Nevertheless, the opposite trend has happened in Cyprus, and could contribute to the success of the Cyprus Scops Owl and the stability of its population. Flint [45] stated that because of rural depopulation and agricultural abandonment, vineyard area has greatly decreased, from 414 km^2^ in 1929, to 288 km^2^ in 1960, to 190 km^2^ in 1999 [46] and to 66 km^2^ in 2015 [47]. For the same reason it is likely that pesticide use within these former vineyards, which all lie within the breeding range of Cyprus Scops Owl, has also decreased or largely ceased. Furthermore, since the middle of the last century there has been a long-term, ongoing, and very large increase in the quality and area of forest and woodland on the island, including in the Paphos Forest. This policy of re-afforestation and the extensive regrowth of forest and woodland on abandoned agricultural land following rural depopulation (e.g., [21,48,49,50,51,52]) has provided extensive suitable habitat for Cyprus Scops Owls, hopefully offsetting any losses in renovated villages.

Denac et al. [36] concluded that the Eurasian Scops Owl faced threats throughout its range in continental Europe, as was also found by Marchesi and Sergio [11], Treggiari et al. [53], and Malle and Probst [54]. They found that agricultural landscapes that were converted to monocultures, and where perches, hedges, and vineyards were removed, adversely affected the foraging capabilities of the species. In Central Italy, parakeets (*Psitaculla krameri*) occupied the nest cavities earlier in the year while the migratory Scops Owls were absent, resulting in a reduction of the breeding density [55]. They recommended that natural cavities should not be filled with any materials in traditional homes and villages, and that nest boxes can be placed where there are no natural cavities, but where suitable foraging habitat exists. This recommendation was also forwarded for the Daito Scops Owl [40] because the species nest in invasive trees and their removal could affect the island population and nest boxes were proven to be a viable alternative.

As is well known, endemic island bird species are especially vulnerable to extinction from anthropogenic environmental change and reduced fitness compared with mainland taxa (e.g., [45,56,57,58,59]). In this context it is noteworthy that one of the other two Cyprus endemic bird species, the widespread and common Cyprus Warbler *Sylvia melanothorax*, is now in serious decline following the recent colonization by the congeneric Sardinian Warbler *S. melanocephala* (e.g., [60,61,62]). Therefore, although we are unaware of any current significant threats to the Cyprus Scops Owl, we recommend that its conservation be prioritized, including further studies, regular monitoring, habitat conservation, and the widespread provision of nest boxes.

## 5. Conclusions

Our study illustrated that the Cyprus Scops Owl did not show a preference for any of the three habitats–rural, forest edge, or forest. Also, we found no differences in the breeding parameters between habitats. However, habitat preference, expressed as nest-box occupancy, was highest at the forest-edge and lowest in the forest. We conclude that nest boxes are a viable alternative to nest cavities in the endemic Cyprus Scops Owl.

## Figures and Tables

**Table 1:** Breeding parameters of the Cyprus Scops Owl *Otus cyprius* in nest boxes located in three types of habitats. Mean values are presented with 95% confidence limits in brackets and sample size. Dates of laying date and hatching day are presented in Julian Days.

Habitat	Laying Date (d)	Clutch Size	No. Day of Incubation	Hatching Day	No. of Hatchlings	No. of Fledglings
Forest	125.9 (123.2–128.7); 47	2.4(2.1–2.6); 47	22.1 (21.4–22.8); 38	147.4 (143.9–150.8); 38	2.0 (1.6–2.3); 47	1.9 (1.6–2.2); 47
Edge	124.0 (120.1–127.8); 20	2.5 (1.9–3.0); 20	22.1 (21.3–23.8); 20	146.1 (142.0–150.1); 20	2.5 (1.9–3.0); 20	2.3 (1.7–2.9); 20
Rural	126.3 (122.0–130.6); 24	2.3 (1.9–2.7); 24	22.7 (21.4–23.9); 20	148.2 (142.9–153.4); 20	2.1 (1.5–2.6); 24	2.0 (1.5–1.5); 24
all	125.6 (123.7–127.5); 91	2.4 (2.2–2.6); 91	22.5 (22.2–22.9); 78	147.2 (144.9–149.5); 78	2.1 (1.8–2.3); 91	2.0 (1.8–2.3); 91

## Data Availability

The data set is archived on Mendeley (http://dx.doi.org/10.17632/yz7dw3bm7t.1).

## Supplementary Materials

The following are available online at https://www.mdpi.com/article/10.3390/ani11061775/s1, Figure S1: Nest box photograph provided to Cyprus Scops Owl Otus cyprius in Paphos Forest (Cyprus) showing exact dimensions.
