# Supplementary material for: Endemic Cyprus Scops Owl Otus cyprius Readily Breeds in Artificial Nest Boxes
Source: Animals (Basel). 2021 Jun 14;11(6):1775. doi: 10.3390/ani11061775 (PMC8232148; doi:10.3390/ani11061775)
Supplement: Supplementary file 1 [file animals-11-01775-s001.zip › animals-1150356-supplementary.pdf]

animals

MDPI

Supplementary Materials

Endemic Cyprus Scops Owl *Otus cyprius* Readily Breeds in Artificial Nest Boxes

Savvas Iezekiel [1], Reuven Yosef [2,*], Constantinos Themistokleus [3], Dimitrios E. Bakaloudis [4], Christos G. Vlachos [4], Andreas Antoniou [5], Eandas Iezekiel [5], Malamati A. Papakosta [6] and Jakub Z. Kosicki [7]

1 Department of Forests, Ministry of Agriculture, Rural Development and Environment, 1414 Nicosia, Cyprus; iezekiel@cytanet.com.cy
2 Ben Gurion University of the Negev—Eilat Campus, P. O. Box 272, 88000 Eilat, Israel
3 Department of Forests, Ministry of Agriculture, Rural Development and Environment, Kedron 1 str., 86400, Panagia, Cyprus; cthemistokleous@fd.moa.gov.cy
4 School of Forestry and Natural Environment, Aristotle University of Thessaloniki, P.O. Box 241, 541 24 Thessaloniki, Greece; debakaloudis@for.auth.gr (D.E.B.); cvlachos@for.auth.gr (C.G.V.);
5 Cyprus Association for the Protection of Avifauna Ioanni Kapodistria 41A, 2321 Nicosia, Cyprus; andrantoni@gmail.com (A.A.); Eandas_iezekiel@hotmail.com (E.I.)
6 Department of Forestry and Management of the Environment & Natural Resources, Democritus University of Thrace, 68200 Orestiada, Greece; mpapakos@fmenr.duth.gr
7 Department of Avian Biology and Ecology, Faculty of Biology, Adam Mickiewicz University, Poznań, ul. Uniwersytetu Poznańskiego 6, 61-614 Poznań, Poland; kubako@amu.edu.pl
* Correspondence: ryosef60@gmail.com; Tel.: +972-537671290

Citation: Iezekiel, S.; Yosef, R.; Themistokleus, C.; Bakaloudis, D.E.; Vlachos, C.G.; Antoniou, A.; Iezekiel, E.; Papakosta, M.A.; Kosicki, J.Z. Endemic Cyprus Scops Owl *Otus cyprius* Readily Breeds in Artificial Nest Boxes. **2021**, *11*, 1775. https://doi.org/10.3390/ani11061775

Academic Editor: Hermann Wagner

Received: 11 March 2021
Accepted: 11 June 2021
Published: 14 June 2021

Publisher's Note: MDPI stays neutral with regard to jurisdictional claims in published maps and institutional affiliations.

Copyright: © 2021 by the authors. Submitted for possible open access publication under the terms and conditions of the Creative Commons Attribution (CC BY) license (http://creativecommons.org/licenses/by/4.0/).

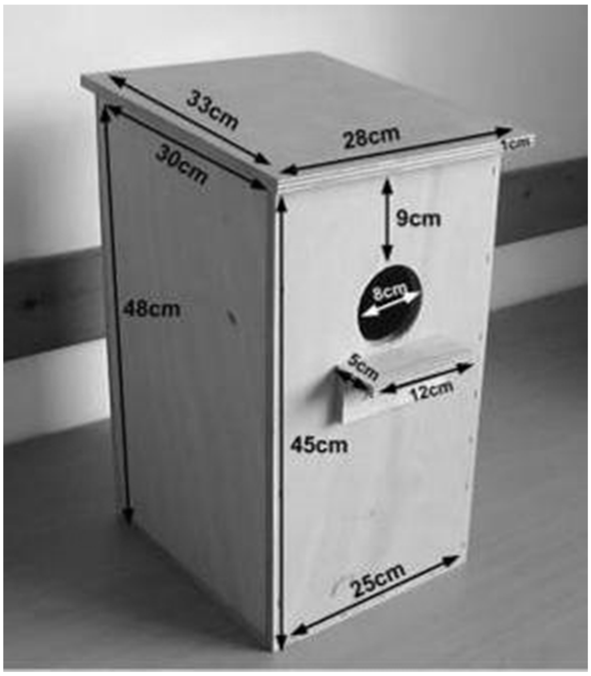

Figure S1. Nest box photograph provided to Cyprus Scops Owl *Otus cyprius* in Paphos Forest (Cyprus) showing exact dimensions.
